# NOG-hIL-4-Tg, a new humanized mouse model for producing tumor antigen-specific IgG antibody by peptide vaccination

**DOI:** 10.1371/journal.pone.0179239

**Published:** 2017-06-15

**Authors:** Yoshie Kametani, Ikumi Katano, Asuka Miyamoto, Yusuke Kikuchi, Ryoji Ito, Yukari Muguruma, Banri Tsuda, Sonoko Habu, Yutaka Tokuda, Kiyoshi Ando, Mamoru Ito

**Affiliations:** 1 Department of Molecular Life Science, Division of Basic Medical Science, Tokai University School of Medicine, Isehara, Kanagawa, Japan; 2 Institute of Advanced Biosciences, Tokai University, Hiratsuka, Kanagawa, Japan; 3 Central Institute for Experimental Animals, Kawasaki, Kanagawa, Japan; 4 Department of Breast and Endocrine surgery, Tokai University School of Medicine, Isehara, Kanagawa, Japan; 5 Department of Hematology and Oncology, Tokai University School of Medicine, Isehara, Kanagawa, Japan; 6 Department of Immunology, Juntendo University School of Medicine, Tokyo, Japan; University of Rhode Island, UNITED STATES

## Abstract

Immunodeficient mice transplanted with human peripheral blood mononuclear cells (PBMCs) are promising tools to evaluate human immune responses to vaccines. However, these mice usually develop severe graft-versus-host disease (GVHD), which makes estimation of antigen-specific IgG production after antigen immunization difficult. To evaluate antigen-specific IgG responses in PBMC-transplanted immunodeficient mice, we developed a novel NOD/Shi-scid-IL2r**γ**^null^ (NOG) mouse strain that systemically expresses the human IL-4 gene (NOG-hIL-4-Tg). After human PBMC transplantation, GVHD symptoms were significantly suppressed in NOG-hIL-4-Tg compared to conventional NOG mice. In kinetic analyses of human leukocytes, long-term engraftment of human T cells has been observed in peripheral blood of NOG-hIL-4-Tg, followed by dominant CD4+ T rather than CD8+ T cell proliferation. Furthermore, these CD4+ T cells shifted to type 2 helper (Th2) cells, resulting in long-term suppression of GVHD. Most of the human B cells detected in the transplanted mice had a plasmablast phenotype. Vaccination with HER2 multiple antigen peptide (CH401MAP) or keyhole limpet hemocyanin (KLH) successfully induced antigen-specific IgG production in PBMC-transplanted NOG-hIL-4-Tg. The HLA haplotype of donor PBMCs might not be relevant to the antibody secretion ability after immunization. These results suggest that the human PBMC-transplanted NOG-hIL-4-Tg mouse is an effective tool to evaluate the production of antigen-specific IgG antibodies.

## Introduction

To develop molecular targeting reagents, an evaluation system of safety and efficacy is essential. While most immune responses can be evaluated in vitro, responses such as specific antibody production require *in vivo* experiments. For in vivo preclinical studies, experimental animals such as rodents and non-human primates have been used. However, because they have numerous species differences, side effects would be overlooked in preclinical studies and occur in clinical studies [1–3]. Moreover, the evaluation of a vaccine response is impossible because rodents lack orthologs of human major histocompatibility complex (MHC) and show low homology among TCR repertoires [4,5]. Thus, these models are insufficient to evaluate human immune responses [6], and eventually it will be necessary to evaluate the efficacy and toxicity of vaccination based on human immunity. Therefore, humanized mice are being explored for the development of new drugs. To date, three principal approaches have been established to generate humanized mice that have been reconstituted with human immune cells: hematopoietic stem cell (HSC)-, fetal bone marrow (BM)/liver/thymus (BLT) tissue, and peripheral blood mononuclear cell (PBMC)-transplanted immunodeficient mice [7–12]. In HSC-transplanted humanized mice, human T cell responses and antigen-specific IgG production are impaired because murine MHC-restricted human T cells fail to interact with human B cells, although HLA-transgenic (Tg) humanized mice show somewhat improved responses [13–15]. Although BLT mice are a better model for developing functional human T and B cells, they present ethical problems that are not a concern in PBMC-transplanted mice [16,17]. Moreover, PBMC-transplanted mice do not require HLA gene introduction because mature T and B cells are engrafted. However, PBMC-transplanted mice develop xenogeneic (xeno) GVHD, resulting in murine illnesses [18,19]. In particular, increased quantities of Th1 cytokines have been observed in an acute GVHD model in humanized mice and in GVHD patients [20–22].

In this study, we hypothesized that if human immune responses in PBMC-transplanted mice shifted to a Th2 phenotype, humoral immunity may be maintained without GVHD. Therefore, we established a novel strain of NOG mice in which human IL-4, a representative Th2 cytokine, is systemically expressed, and examined whether this mouse strain can support antigen-specific human antibody production in response to HER2 peptide vaccination.

## Materials and methods

### Ethical approval

Human PBMCs from healthy volunteer donors were obtained after receiving written informed consent based on Institutional Review Board-approved protocols according to institutional guidelines. This work was approved by the Tokai University Human Research Committee (12R-002) and Central Institute for Experimental Animals (CIEA) Human Research Committee (08–01). These studies were conducted in accordance with the Declaration of Helsinki protocols and all Japanese federal regulations required for the protection of human subjects. Use of immunodeficient mice for xenotransplantation studies was approved in compliance with the Guidelines for the Care and Use of Laboratory animals, and all animal studies were approved by the committees of CIEA and the Tokai University School of Medicine.

### Generation of NOG-IL-4-Tg mice

Non-obese diabetic (NOD/Shi) mice were purchased from CLEA Japan, Inc. (Tokyo, Japan), and NOD/Shi-scid-IL2r**γ**null (NOG; formal name, NOD.Cg-*Prkdc*^*scid*^*il2r***γ**^*tm1Sug*^ /ShiJic) mice were maintained in CIEA under specific pathogen-free (SPF) conditions. To generate the NOG-hIL-4-Tg mice, human IL-4 cDNA was replaced with the β-galactosidase gene from the pCMVβ vector (Clontech, Inc., Mountain View, USA). The vector was digested with XhoI restriction enzyme, and the linearized fragment (~4.2 Kb) was microinjected into pre-nuclear-stage fertilized eggs obtained by mating NOD/Shi and NOD-IL-2Rγ^*null*^ mice.

The offspring with the inserted transgene were selected by PCR amplification of a 561-bp fragment with the primer set 5’-cccgggatcgttagcttctcctgataaa-3’ and 5’-gcggccgctattcagctcgaacactttgaat-3’, and the hIL-4 concentration in the sera was measured by ELISA (BD OptEIA^TM^, BD Biosciences, San Diego, CA). For ELISA, peripheral blood (PB) was collected from the orbital venous plexus of 4- to 6-week-old mice using heparin (Novo-heparin; Mochida Pharmaceutical Co., Tokyo, Japan)-coated capillaries (Drummond Scientific, Broomall, PA) under anesthesia. Founder mice were further backcrossed with NOG mice to obtain NOG-hIL-4-Tg mice (formally, NOD.Cg-*Prkdc*^*scid*^*il2rγ*^*tm1Sug*^ *Tg (CMV-IL4)3-2Jic*/Jic).

As control mouse DNA, primers of M06987F:5’-gagataccaggagcccttcc-3’ and M06987R:5’-cagactctgcaagcctctca-3’ were used. For the hIL-4 DNA, primers of hIL-4F: 5’-cccgggatcgttagcttctcctgataaa-3’ and hIL-4R: 5’-gcggccgctattcagctcgaacactttgaat-3’ were used. NOG and NOG-hIL-4-Tg mice were housed under SPF conditions in the animal facility located at CIEA or Tokai University School of Medicine during the experiments.

### Tissue-specific expression of hIL-4 mRNA

Mice were anesthetized with 20% isoflurane, 40 ml of heparinized PBS was injected via the left ventricle, and the right atrium was cut for exsanguination. After perfusion, the organs were excised, minced and stored in TRIzol (Life Technologies, Carlsbad, CA, USA). RNA was extracted, and mRNA expression was examined by RT-PCR as mentioned below. For mouse β-actin cDNA primers, β-actin F: 5’-atgaggtagrctgtctgtcaggt-3’ and β-actin R: 5’-atggatgacgatatcgct-3’ were used. For human IL-4 cDNA primers, hIL-4F: 5’-ctgcaaatcgacacctatta-3’ and hIL-4R: 5’-gatcgtctttagcctttc-3’ were used.

### Preparation and transplantation of human PBMCs

A total of 7.5 ml PB from healthy donors was drawn into Vacutainer ACD tubes (NIPRO Corporation, Japan, Osaka) containing heparin. The collected PB was immediately placed in 10 ml of Ficoll-Hypaque (SIGMA-ALDRICH, UK, London), and mononuclear cells were isolated by density centrifugation (500 × *g*, 30 min, 20°C). The cells were washed with phosphate-buffered saline (PBS) for 5 min at 300 × *g*, 4°C. Doses of 2.5 to 5x10^6^ PBMC were transplanted intravenously into 8- to 12-week-old NOG or NOG-hIL-4-Tg mice. For the GVHD assay, the mice were irradiated (2.5 Gy), and 1 day after irradiation, the PBMCs were transplanted. The body weights of the individual mice were measured weekly. The number of mice transplanted depended on the amount of available material, particularly the healthy donor (HD) PBMCs.

### Analysis of engrafted human cells in PBMC-NOG-hIL-4-Tg mice

At 6 to 8 weeks after transplantation, the mice were euthanized for analysis. PB was collected in the presence of heparin via retro-orbital bleeding under inhalation anesthesia at 4 weeks after transplantation. The mice were sacrificed and analyzed for T and B cell development and antibody production after 28 days. For the GVHD assay, PB was collected once a week up to 10 weeks after transplantation. Engraftment and differentiation of human cells in the BM, spleen and PB were analyzed by flow cytometry (FCM) staining with anti-human antibodies as described below. MNCs were prepared, and the reconstitution rate of human cells was determined based on hCD45 expression in the lymphoid-gated fraction.

To assess Th1/Th2 cytokine production, CD4 T cells were purified using the human CD4+ T Cell Isolation Kit 2 (Miltenyi Biotec) from human PBMCs and transplanted into NOG and NOG-hIL-4-Tg mice (1x10^6^) at 1 day after irradiation (2.5 Gy). The transplanted CD4+ T cells were collected from the spleen at 2 weeks after transplantation and analyzed by FCM following intracellular staining.

Fluorochrome-conjugated anti-human monoclonal antibodies (mAbs) were used to identify human immune cells. The cells were incubated with appropriate dilutions of fluorescently labeled mAbs for 15 min at 4°C and were then washed with PBS containing 1% (w/v) BSA. The cells were analyzed using FACS Fortessa, Verse, or Canto (BD Bioscience, Franklin Lakes, NJ). For each analysis, the live gate with white blood cells or lymphocytes was further gated based on hCD45 expression. The mouse anti-human mAbs used in this study are listed in S1 Table.

Intracellular staining was performed as follows. Two weeks after transplantation, spleen CD4+ T cells were purified as mentioned above, and 1 x 10^6^ cells were cultured in RPMI1640 medium with 10% fetal bovine serum (FBS) containing phorbol 12-myristate 13-acetate (PMA; 50 ng/mL) and ionomycin (1 μg/mL) for 4 h in 24-well culture plates in the presence of Brefeldin A (1:1,000 dilution). Cells were stained with fluorochrome-conjugated mAbs as shown in S1 Table and analyzed by FCM according to the manufacturer’s instructions (Human Th1/Th2 Flow Panel Kit Ready-Set-Go; eBioscience, San Diego, CA).

### Peptide immunization

A CH401 peptide, which includes the epitope sequence of the anti-HER2 mAb, was determined using MAP-peptides with a partial amino acid sequence of HER2/neu [23]. The peptide was synthesized using a Rink amide resin (0.4–0.7 mmol/g), an ACT357 peptide synthesizer (Advanced Chemtech, Louisville, KY) and a multiple antigen peptide named CH401MAP, a 20-mer peptide of the HER2 molecule that was synthesized as an antigen peptide. HER2 peptide or keyhole limpet hemocyanin (KLH; Sigma-Aldrich co. St. Louis, MO) was emulsified with CFA (Wako Pure Chemical Industries, Ltd, Osaka, Japan) (50 μg/head, 100 μl 1:1/v:v) and administered to the PBMC-NOG-hIL-4-Tg mice intraperitoneally. For the negative control, an equal volume of PBS was emulsified and injected into PBMC-NOG-hIL-4-Tg mice transplanted with the same HD PBMCs. Boosters were performed using IFA (Wako Pure Chemical Industries, Ltd) at 2 weeks after the first immunization. Two weeks after the booster, the mice were sacrificed for analyses.

### Peptide and MHC binding affinity simulation

Computer algorithms accessible from internet databases, namely SYFPEITHI developed by Rammensee et al. [24] (http://www.syfpeithi.de/); epitope prediction based on a database for HLA class I and class II, namely, BioInformatics and Molecular Analysis Section (BIMAS) developed by Parker et al. [25] (http://www-bimas.cit.nih.gov/molbio/hla_bind/) for HLA class I and class II prediction; and the Immune Epitope Database (IEDB) and analysis resource developed by Vita et al. [26] (http://www.immuneepitope.org/) for HLA class II prediction, were used to predict CH401 MAP peptide binding affinity.

### HLA typing analysis

The remaining volume of the HD blood (1.5 ml) was used for HLA typing analysis. The genotypes of the HLA-A, B, and DRB1 alleles were analyzed using the PCR-SSOP Luminex method using LABType SSO (One Lambda Inc., Canoga Park, CA, USA), a reverse SSO DNA typing system, according to the manufacturer’s instructions. A flow analyzer, LABScanTM 100, was used to determine the fluorescence intensity of phycoerythrin on each microsphere. Determination of the HLA allele was based on the reaction pattern compared with patterns associated with published HLA allele sequences (http://www.hla.or.jp/), which included all HLA allele types that are currently known.

### ELISA

The level of human IL-4 protein was measured using the Human IL-4 ELISA Set BD OptEIA^TM^ (BD Biosciences) according to the manufacturer’s instructions. The protocol for specific IgG antibody detection has been previously described [4]. Briefly, micro-wells of microtiter plates (Sumiron, Tokyo, Japan) were coated with CH401MAP peptide or KLH (1 μg/ml) diluted in carbonate buffer (pH 9.5), and the antigens were adsorbed to microtiter plates overnight at 4°C. The wells were washed with PBS-Tween (0.05% v/v) and blocked with 3% BSA-PBS at room temperature (RT) for 2 h. After three washes with PBS-Tween, 10-fold serial dilutions of mouse plasma were added to the wells and incubated for 2 h at RT. The plates were washed three times before addition of biotin-conjugated mouse anti-human IgG mAb (BD Pharmingen, San Diego, USA) (1:3,000). After a 2-h incubation at 37°C, the plates were washed 3 times, followed by the addition of streptavidin-horseradish peroxidase (1:50,000 v/v; BD Pharmingen). The plates were incubated for 1 h at RT, and unbound conjugates were removed by washing. EIA substrate kit solution (Bio-Rad Laboratories, Hercules, CA, USA) was then added to each well. The reaction was stopped with 10% HCl, and the absorbance was measured at 450 nm.

### Histochemical analysis

NOG-IL-4-Tg mouse spleen tissues were fixed with 20% buffered formalin (Wako Pure Chemical Industries, Ltd) and embedded in paraffin. A paraffin block was micro-sectioned and de-paraffinized, and post-fix tissue sections on glass slides were stained with hematoxylin and eosin (HE).

For plasma/plasmablast cell detection, BM and spleen cells were sorted by magnetic bead depletion of CD3+ cells, and cytospin slides were prepared using a Cytospin 4 Cytocentrifuge (Thermo Scientific, Waltham, MA) at 500 rounds per minute for 5 min. To observe the cellular morphology, May-Grunwald-Giemsa staining was performed. May-Grunwald Stain Solution (Wako co. Ltd, Tokyo, Japan) and Giemsa’s Azur Eosin Methylene Blue Solution (Merck, Darmstadt, Germany) were used according to the manufacturer’s instructions.

### Statistics

Statistical analysis was performed with Microsoft Excel (Microsoft, Redmond, WA). The data are shown as the mean ± SD. Significant differences between groups were determined by two-sided Student’s *t*-test analysis.

## Results

### Establishment of NOG-IL-4-Tg

**Fig 1:**
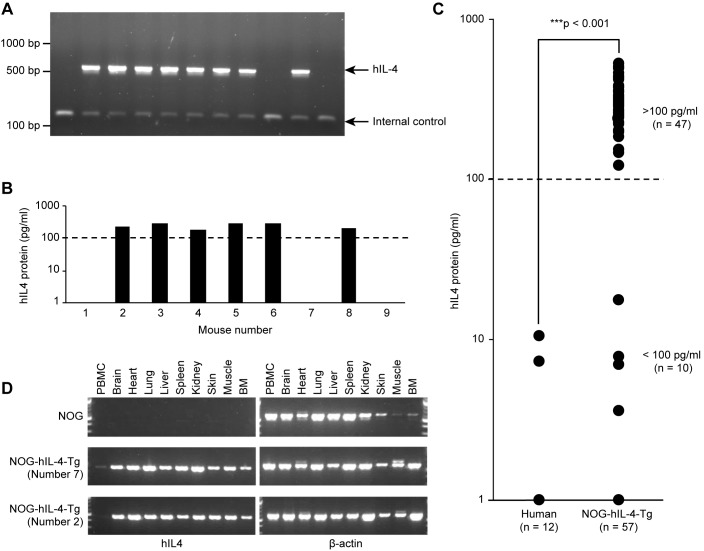
Generation of NOG-hIL-4-Tg mice. **A,** Genotyping of NOG-hIL-4-Tg mice. Human IL-4-specific bands (561 bp) were detected along with the internal control (151 bp). PC: positive control. These results confirmed the gene transfer. NC: negative control. Conventional NOG mice were used. **B,** ELISA of NOG-IL-4-Tg plasma corresponding to the mice submitted for genotyping. The mice producing more than 100 pg/ml hIL-4 (higher than the broken line) were selected and used for the assays. A representative assay is shown. **C,** Comparison of hIL-4 protein levels in HD plasma (n = 12) and NOG-hIL-4-Tg mice (n = 57). The broken line shows the 100 pg/ml level. The high hIL-4 (n = 47) and low hIL-4 groups (n = 10) are shown. **D**, Tissue-specific expression of hIL-4 mRNA. Representative data of 1 NOG and 2 NOG-hIL-4-Tg mice are shown. Human IL-4 specific bands (449 bp) were detected along with β-actin (569 bp).

After microinjection of human IL-4 cDNA into NOD, (NOD x NOG) F1 or NOG mouse embryos, we obtained 3 Tg founders (#3, #17, #26) from 93 weanlings. The serum hIL-4 concentrations were 73.9 pg/ml in founder #3, 321.5 pg/ml in founder #17 and 656.5 pg/ml in founder #26 (S1 Fig). We used the Tg mouse line from founder #17 because it demonstrated an intermediate level of hIL-4, and the inserted transgene was stably transmitted to its progeny. We verified the transmission of the hIL-4 gene to the progeny by PCR and secretion of the protein by ELISA, as shown in Fig 1A and 1B. NOG-hIL-4-Tg mice could be clearly differentiated into two groups based on hIL-4 expression. The high expression group (>100 pg/ml), which included more than 80% of the hIL-4 gene-positive mice, secreted 10- to 100-fold the amount of hIL-4 in comparison to HD (6 pg/ml on average in our results), as shown in Fig 1C. All of the transgene-positive mice in both the high and low (<100 pg/ml) expression groups were healthy, and no obvious illness was observed in the absence of treatment. Most of the organs except PBMCs expressed hIL-4 mRNA, irrespective of the plasma hIL-4 levels (Fig 1B and 1D). This result suggested that undetectable quantities of hIL-4 might be secreted throughout the system. We used the high hIL-4 expression group (>100 pg/ml) for human PBMC transplantation in this study.

### GVHD suppression in human PBMC-transplanted NOG-IL-4-Tg

**Fig 2:**
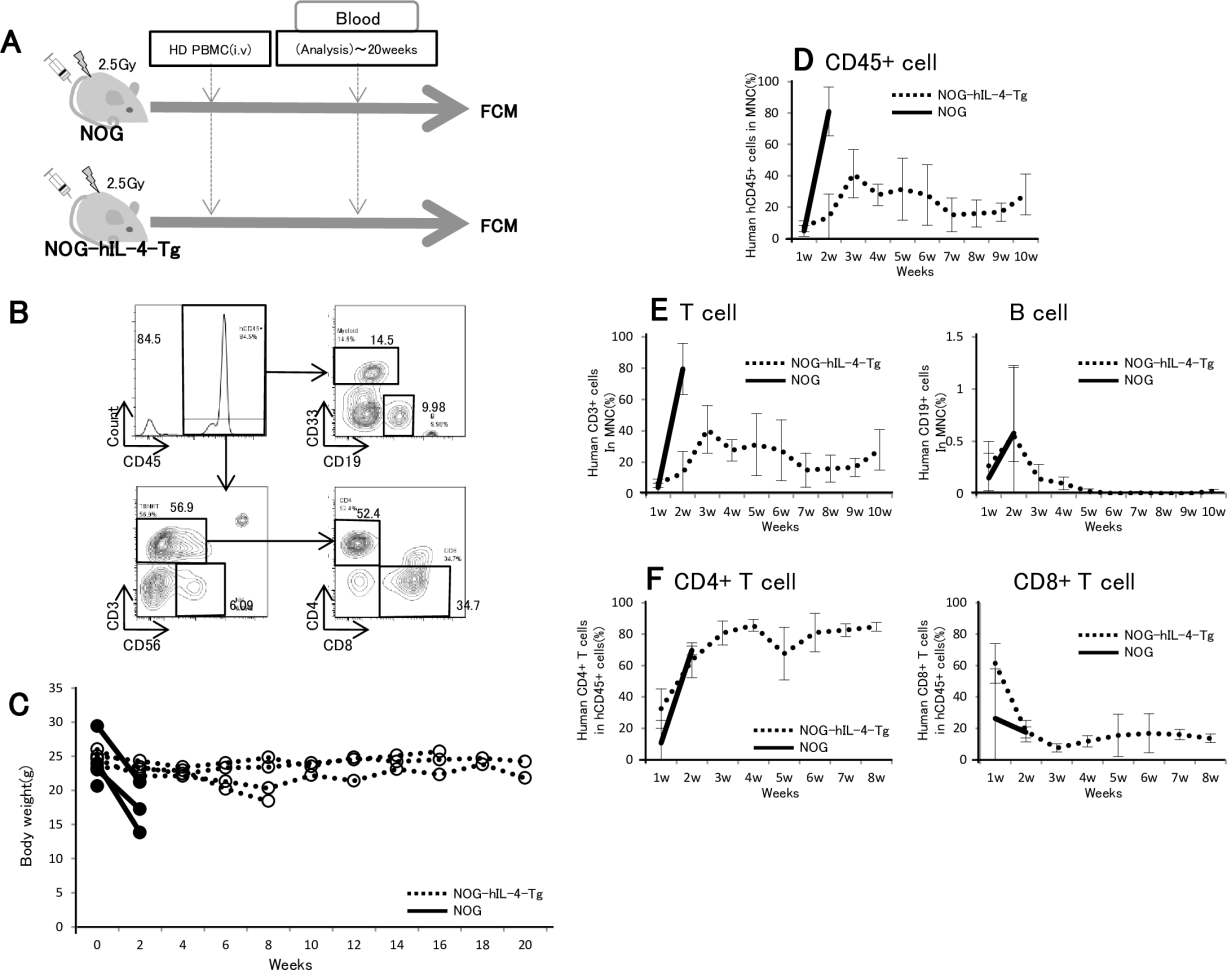
Decreased GVHD symptoms in PBMC-NOG-hIL-4-Tg mice. **A,** Experimental protocol for hPBMC transfer to NOG-hIL-4-Tg and conventional NOG mice. Doses of 2.5x10^6^ PBMCs were intravenously transferred into NOG-hIL-4-Tg and NOG mice 1 day after 2.5 Gy irradiation. After cell transfer, human lymphocytes were analyzed by FCM. **B,** Typical flow cytometric pattern of PBMC-transplanted mice. The CD45+ fraction was further analyzed for CD33+CD19- myeloid cells, CD19+ B cells, CD56+CD3- NK cells, CD3+CD4+ T cells and CD3+CD8+ T cells. The data presented in these panels show the percentages of these cell fractions. **C,** Suppression of GVHD in NOG-hIL-4-Tg mice. After HD PBMC transplantation, the body weights of NOG-hIL-4 Tg and NOG mice were measured biweekly for 20 weeks. Open circles and dashed lines: individual NOG-hIL-4-Tg mice; closed circles and solid lines: individual NOG mice. **D-E,** From 1 to 10 weeks after HD PBMC transplantation, the engraftment rates of human PBMCs were analyzed by FCM. **D,** Human CD45+ cells (%) in mononuclear cells (MNCs). **E,** Left panel; human CD3+ cells (%) in MNCs, right panel; human CD19+ cells (%) in MNCs. **F,** From 1 to 8 weeks after HD PBMC transplantation, the engraftment rates of human PBMCs were analyzed. Left panel; CD4+ T cells and CD8+ T cells in human CD3+ cells (%) in hCD45+ cells (%) of the mice, right panel; human CD4+ T cells and CD8+ T cells in human CD3+ cells (%) in hCD45+ cells (%) of the mice. Dotted lines: NOG-hIL-4-Tg mice; solid lines: NOG mice.

We examined whether xeno-GVHD is suppressed in PBMC-NOG-hIL-4-Tg compared with the conventional NOG mice. In this system, we irradiated the mice to increase the sensitivity to GVHD and transplanted human PBMCs intravenously as shown in Fig 2A. The PBMC cellularity was analyzed by FCM as shown in Fig 2B. All the PBMC-NOG died within 2 weeks after PBMC transplantation, whereas 50% of the NOG-hIL-4-Tg survived for more than 20 weeks (Fig 2C). In these survivor mice, body weight did not decrease during the 20-week period (Fig 2C). CD45+ cells and CD45+CD3+ T cells were maintained after a transient increase, while CD45+CD19+ B cells gradually decreased after transplantation (Fig 2D and 2E). A high ratio of CD4+ T cells was maintained for more than 8 weeks in NOG-hIL-4-Tg mice (Fig 2F). These results indicate that severe GVHD was effectively suppressed by hIL-4 in hPBMC-transplanted mice.

### Characteristics of Human PBMCs in NOG-IL-4-Tg

**Fig 3:**
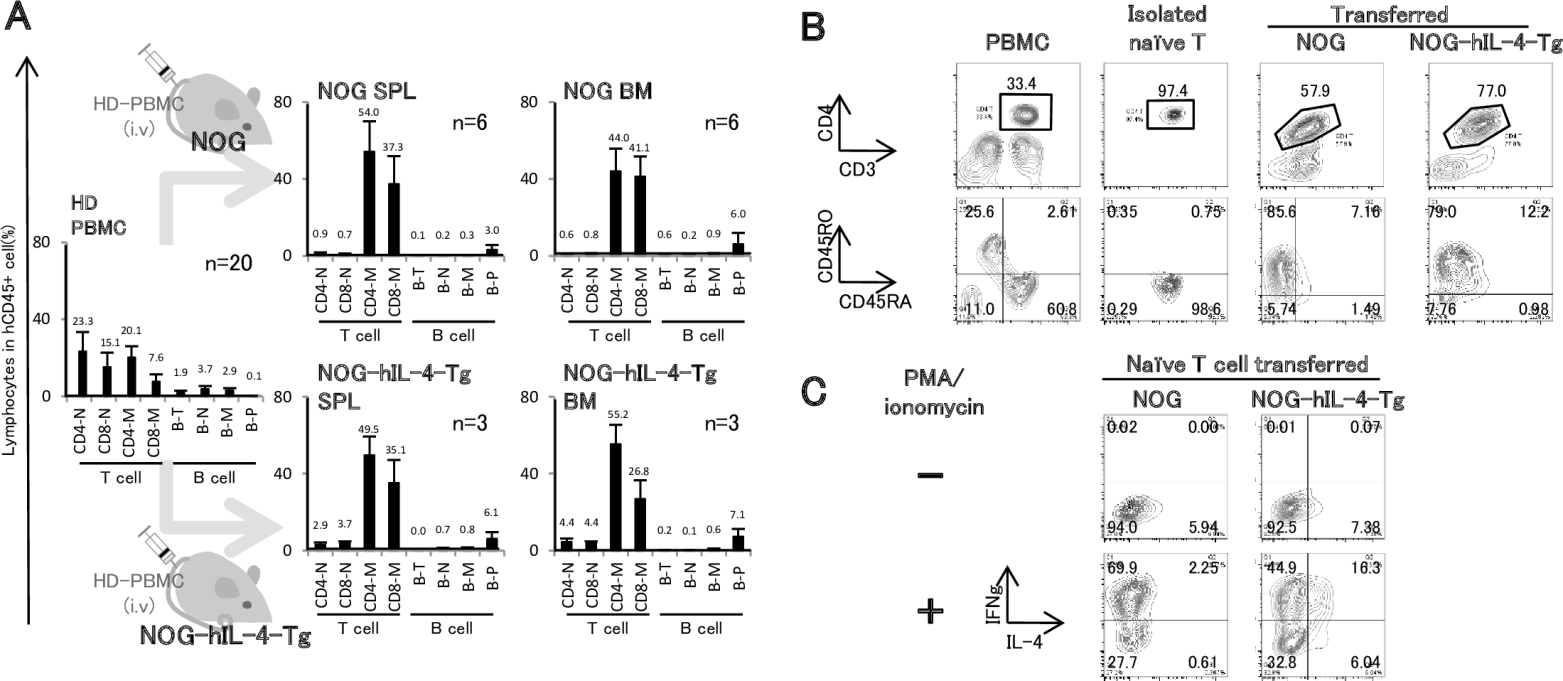
Comparison of engrafted human lymphocytes in NOG-hIL-4-Tg and conventional NOG mice. **A,** Cellularity in HD PBMC-transferred NOG or NOG-hIL-4-Tg mice. NOG-hIL-4-Tg and NOG mice were transplanted with PBMCs (5x10^6^) from the same HD. After 4 weeks, the ratios of each T or B cell subset in BM or spleen obtained from PBMC-NOG (n = 6) and PBMC-NOG-hIL-4-Tg mice (n = 3) were analyzed by FCM. HD PBMCs (n = 20) are shown as a control. CD4-N; naïve CD4+ T cells, CD8-N; naïve CD8+ T cells, CD4-M; memory CD4+ T cells, CD8-M; memory CD8+ T cells, B-T; transitional B cells, B-N; naïve B cells, B-M; memory B cells, B-P; plasmablast/plasma cells. The mean ± SD is shown with the percentage score above each bar. **B.** Human naïve CD4+ T cells were purified and transferred to both NOG mice (n = 5) and NOG-hIL-4-Tg mice (n = 8). The left 4 panels show the typical expression profiles of CD45RA, CD45RO, CD3 and CD4 among purified naïve CD4+ T cells. MNCs were obtained from PBMC-NOG or PBMC-NOG-hIL-4-Tg mice, and the human CD4+ T cells were analyzed by FCM as shown in the 4 right panels. The lower panels were all gated on the upper gates surrounded by the lines. **C,** Isolated human CD4+ T cells were stimulated with PMA and ionomycin, and the expression levels of human IL-4 and IFN-γ were analyzed by FCM. Typical patterns are shown.

Next, we investigated the characteristics of the engrafted human T and B cells in NOG-hIL-4-Tg mice. The onset of GVHD was delayed in order to compare T and B cellularity and the amount of antigen-specific IgG antibodies detected in HD PBMC-transplanted NOG and NOG-hIL-4-Tg mice. Therefore, twice the amount of hPBMCs was transplanted into NOG-hIL-4-Tg or conventional NOG mice without irradiation (Fig 3A). At 4 weeks after transplantation, typical flow cytometry patterns of T and B cells were observed (S2 Fig). The mean ratios of the T and B cell subsets derived from PBMC-NOG and PBMC-NOG-hIL-4-Tg mice were compared along with those of the original PBMCs (Fig 3A). In PBMC-NOG BM, the frequencies of both memory CD4+ and CD8+ T cells were prominently increased at 4 weeks after transplantation. In contrast, in PBMC-NOG-hIL-4-Tg mice, the ratio of memory CD8+ T cells was lower than that of memory CD4+ T cells. Very few naïve CD4+ and CD8+ T cells were observed in both groups of mice. CD4+ T cells from PBMC-NOG-hIL-4-Tg mice expressed high levels of PD-1 but very low levels of CD25 (S3A Fig), suggesting that these T cells contained exhausted T cells and CD25+PD-1+ Tfh-like cells [27,28]. In both PBMC-NOG and NOG-hIL-4-Tg mice, the plasma cell ratio was higher than that observed in the original PBMC population (Fig 2A). Whereas most of the remaining B cells in the PBMC-NOG mice were CD21-CD24- cells, indicating B cell exhaustion, the frequency of CD21+CD24+CD27+ conventional memory B cells increased in the PBMC-NOG-hIL-4-Tg mice, as shown in S3B Fig [29].

As shown in Fig 3B, naïve CD4+ T cells were purified and transplanted into NOG or NOG-hIL-4-Tg mice. Most of the engrafted CD4+ T cells in naïve T cell-transferred NOG mice expressed IFN-γ protein, whereas very few IL-4 protein-producing cells were detected after stimulation with PMA and ionomycin in vitro (Fig 3C). However, NOG-hIL-4-Tg-derived CD4+ T cells expressed significant amounts of IL-4 after stimulation. Without stimulation, similar levels of IL-4 positive cells were observed both in NOG and NOG-IL-4-Tg, suggesting that these cells were activated in the xeno-environment. These results demonstrated that hIL-4 induced Th2 cytokine production. Collectively, the NOG-hIL-4-Tg mice provided a Th2-conducive environment for human PBMCs.

### Specific IgG production by CH401MAP immunization

**Fig 4:**
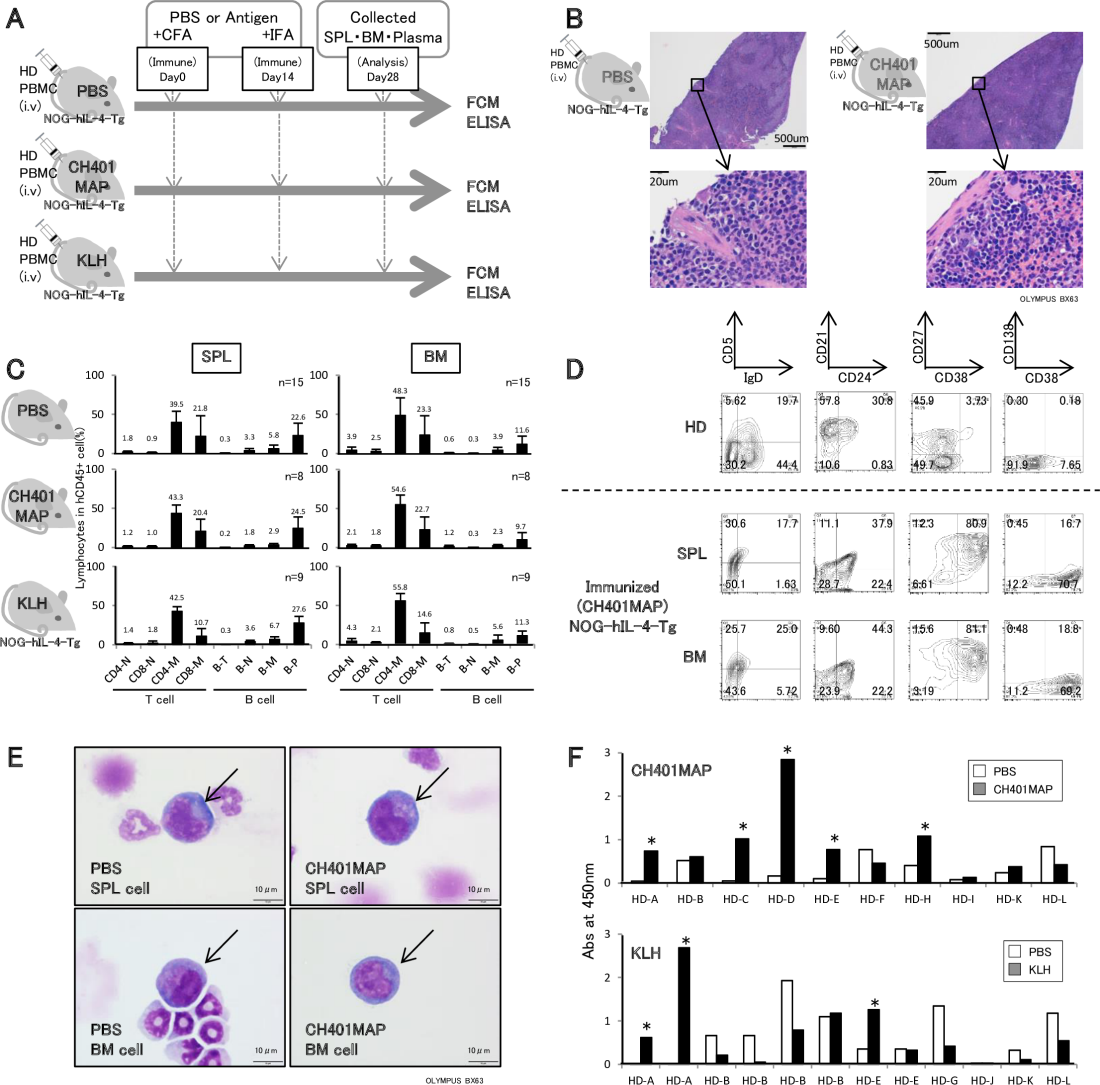
The humoral immunity of transplanted NOG-hIL-4-Tg mice induced by antigen immunization. **A,** Experimental protocol for immunization of PBMC-NOG-hIL-4-Tg mice. After transplantation with HD PBMCs, mice were immunized with CH401MAP or KLH emulsified in complete Freund’s adjuvant (CFA). As a negative control, PBS was emulsified with the adjuvant. After 14 days, CH401MAP or KLH was boosted with incomplete Freund’s adjuvant (IFA). Finally, after 28 days, the mice were sacrificed and analyzed. **B,** HE staining of NOG-IL-4-Tg spleens. Upper panels; the spleen sections of control PBS and CH401MAP-immunized PBMC-NOG-hIL-4-Tg mice. Lower panels; enlarged view of each spleen section. **C,** Cellularities of human lymphocytes in the spleen and BM of immunized PBMC-NOG-hIL-4-Tg mice. The ratio of each lymphocyte subset among CD45+ cells (%) is shown in the panels. Upper left: PBS and adjuvant-injected spleen; upper right: PBS and adjuvant-injected BM; middle left: CH401MAP and adjuvant-injected spleen; middle right: CH401MAP and adjuvant-injected BM; lower left: KLH and adjuvant-injected spleen; lower right: KLH and adjuvant-injected BM. The mean ± SD is shown with the percentage score above each bar. **D,** Typical B cell profiles of the lymphocytes in HD PBMCs and CH401MAP-immunized PBMC-NOG-hIL-4-Tg mouse spleen and BM cells. Upper panels; HD patterns. Middle panels: PBMC-NOG-hIL-4-Tg spleen cells; Lower panels: PBMC-NOG-hIL-4-Tg BM cells. All density plots were gated on CD45+CD19+ cells. **E,** Morphology of plasmablast/plasma cells in the BM or spleen of PBMC-NOG-hIL-4-Tg mice. After depletion of CD3+ T cells, Giemsa staining was performed. Upper panels: spleen; lower panels: BM. The plasmablast/plasma cells with typical morphology are shown with arrows. **F,** Plasma samples from immunized NOG-hIL-4-Tg mice were collected, and antigen-specific IgG was measured by ELISA. Upper panel: CH401MAP-immunized mouse plasma (n = 10); lower panel: KLH-immunized mouse plasma (n = 12). The PBMCs of HD-A, HD-B, HD-K and HD-L were transplanted into 3 mice, and PBS/CFA, CH401MAP/CFA and KLH/CFA were injected into each mouse. Therefore, the same control PBS/CFA data were used for titer comparisons between anti-CH401MAP IgG and anti-KLH IgG treatment with PBS controls. Thus, the total PBS control mouse number was 18. Open bars: mouse treated with PBS/CFA (PBS). Closed bars: CH401MAP/KLH-immunized mouse. The figure above each bar shows the accurate absorbance at 450 nm. Asterisks; indicate CH401MAP-immunized serum for which the reactivity was two-fold higher than the control sera in mice transplanted with the same donor PBMCs.

To confirm the usefulness of these mice for monitoring the vaccine effects, PBMC-NOG-hIL-4-Tg mice were immunized with CH401MAP, a 20-mer HER2 peptide, to induce the production of HER2-specific antibodies [23]. As negative and positive controls, equal volumes of PBS or KLH solution were administered to the NOG-hIL-4-Tg mice transplanted with PBMCs from the same donor (Fig 4A).

Histochemical analysis showed that B cell follicles did not appear in the spleens of PBMC-NOG-hIL-4-Tg mice with or without antigen immunization (Fig 4B), suggesting that affinity maturation did not occur in these mice. Similar to Fig 3A, memory CD4+ T cells were dominant, and the ratio of naïve T cells decreased (Fig 4C). However, the cellularity was not different among the PBS, CH401MAP and KLH immunization groups (Fig 4C and S5 Fig). High ratios of plasma/plasmablast B cells were observed in the spleens of all three groups. Compared to NOG-IL-4-Tg, the number and the ratio of plasma cells in the spleen tended to be low in NOG mice after immunization (S5 Fig).

Next, we examined CD138 expression in CD38+CD27+ cells because CD138 is a plasmablast/plasma cell marker [30]. CD138 expression on CD38+CD27+ cells was very low, suggesting incomplete maturation (Fig 4D). However, cells with an abundant basophilic cytoplasm and round, eccentric nuclei with coarse chromatin and prominent perinuclear hofs, indicative of a typical plasma cell morphology, were observed in both the BM and spleen (Fig 4E). Plasmablasts usually accumulate in human BM, but they accumulated to a much greater extent in the spleen compared with the BM in PBMC-NOG-hIL-4-Tg mice (Fig 4C).

The level of specific IgG in PBMC-NOG-hIL-4-Tg plasma collected from immunized mice was measured by ELISA. As shown in Fig 4F, plasma samples from 6 out of 10 independent HDs showed CH401MAP-specific IgG production. We determined the HD HLA haplotypes and compared the affinity for the peptide predicted using three major algorithms: SYFPEITHI, BIMAS and IEDB. We found that the HD with the highest antibody production possessed an intermediate DRB-1 score, suggesting efficient presentation of the peptide by HLA class II (Table 1). However, the PBMCs containing HLAs with low scores also secreted significant amounts of specific IgG (Fig 4F and Table 1). As shown in S6 Fig, we could not observe a correlation of human IL-4 concentration and CH401MAP-specific IgG amounts in mouse sera (R^2^ = 0.04683, S6 Fig). Moreover, in 3 out of 12 KLH-immunized mice, antigen-specific IgG was detected in the plasma. As the control (PBS) plasma tended to show high cross-reactivity, the frequencies of IgG-positive mice may have been underestimated.

**Table 1:** The list of classical HLA types of HDs.

Donor No.	HLA-A			
	allele	score		
		SYFPEITHI	BIMAS	IEDB
HD-A	A*11:01	17	2.4	0.5–98.0
	A*24:02	12	-	2.5–100
HD-B	A*11:01	17	2.4	0.5–98.0
	A*24:02	12	-	2.5–100
HD-C	A*24:02	12	-	2.5–100
	-	-	-	-
HD-D	A*02:06	0	-	3.3–100
	A*03:01	ND	ND	0.35–100
HD-E	A*02:01	20	2.8	0.7–95
	A*26:02	ND	ND	-
HD-F	A*24:02	12	-	2.5–100
	-	-	-	-
HD-G	A*11:01	17	2.4	0.5–98.0
	A*33:03	ND	ND	1.4–99
HD-H	A*02:01	20	2.8	0.7–95
	A*24:02	12	-	2.5–100
HD-I	A*26:01	0	-	0.9–99
	A*33:03	ND	ND	1.4–99
HD-J	A*24:02	12	-	2.5–100
	-	-	-	-
HD-K	A*24:02	12	-	2.5–100
	A*31:01	8.7	20.5	2.0–90
HD-L	A*24:02	12	-	2.5–100
	A*33:03	ND	ND	1.4–99
Donor No.	HLA-B			
	allele	score		
		SYFPEITHI	BIMAS	IEDB
HD-A	B*15:01	11	-	8.8–100
	B*35:01	-	1	13–100
HD-B	B*15:01	11	-	8.8–100
	B*55:02	ND	ND	ND
HD-C	B*52:01	-	13.2	5.2–97
	B*54:01	-	-	ND
HD-D	B*35:01	-	1	13–100
	B*39:01	24	27	0.4–100
HD-E	B*15:01	11	-	8.8–100
	B*40:01	14	-	2.6–100
HD-F	B*52:01	-	13.2	5.2–97
	-	-	-	-
HD-G	B*44:03:01	0	15	2.7–98
	B*54:01	-	-	ND
HD-H	B*15:18	ND	ND	ND
	B*07:02	13	-	12–100
HD-I	B*40:02	0	-	1–96
	B*44:03:01	0	15	2.7–98
HD-J	B*52:01	-	13.2	5.2–97
	-	-	-	-
HD-K	B*07:02	13	-	12–100
	B*15:01	11	-	8.8–100
HD-L	B*07:02	13	-	12–100
	B*44:03:01	0	15	2.7–98
Donor No.	DRB1			
	allele	score		
		SYFPEITHI	BIMAS	IEDB
HD-A	DRB1*01:01	18	-	19.65–77.45
	DRB1*04:06	ND	ND	25.34–56.36
HD-B	DRB1*04:06	ND	ND	25.34–56.36
	DRB1*14:05	ND	ND	14.88–25.79
HD-C	DRB1*01:01	18	-	19.65–77.45
	DRB1*15:02	0	4.64	4.64–24.45
HD-D	DRB1*04:05	0	-	20.42–44.97
	DRB1*08:02	0	2.76	2.76–9.43
HD-E	DRB1*04:06	ND	ND	25.34–56.36
	DRB1*14:06	ND	ND	17.26–22.80
HD-F	DRB1*14:54	ND	ND	9.42–22.12
	DRB1*15:02	0	4.64	4.64–24.45
HD-G	DRB1*04:05	0	-	20.42–44.97
	DRB1*13:02	0	2.4	2.40–67.99
HD-H	DRB1*08:03	0	-	14.44–27.23
	DRB1*12:02	ND	ND	19.50–25.04
HD-I	DRB1*13:02	0	2.4	2.40–67.99
	DRB1*16:02	ND	ND	2.40–67.99
HD-J	DRB1*14:54	ND	ND	9.42–22.12
	DRB1*15:02	0	4.64	4.64–24.45
HD-K	DRB1*01:01	18	-	19.65–77.45
	DRB1*14:54	ND	ND	9.42–22.12
HD-L	DRB1*01:01	18	-	19.65–77.45
	DRB1*13:02	0	2.4	2.40–67.99

The classical HLA A, B and DRB1 types of HD-A to HD-L are shown in the panel. The scores indicate the binding affinity of CH401MAP peptide to each HLA type estimated by SYFPEITHI, BIMAS and IEDB.

We immunized 5 NOG mice: 2 control (FCA only) and 3 immunized with CH401MAP. Two HDs were enrolled, and PBMCs were used. As shown in S7 Fig, one CH401MAP-immunized mouse died from GVHD before analysis (day 13 after the booster). Compared to the PBS control, no enhancement of specific antibodies was observed in these mice. The CH401MAP-crossreactive IgG level was significantly elevated for HD32, but no enhancement was observed after immunization. Therefore, the NOG mice might produce CH401MAP-crossreactive immunoglobulins, but the immunization might not enhance the production of CH401MAP-specific antibodies (S7 Fig).

These results suggest that B cell function is maintained in PBMC-NOG-hIL-4-Tg and that antigen-specific antibody production can be monitored by this system irrespective of the HLA type.

## Discussion

In this study, we established a novel NOG mouse strain in which human IL-4 is systemically expressed and developed a specific IgG antibody production system using reconstituted human PBMCs without GVHD. As a result, human T cells and B cells survived over 4 weeks of transplantation. Compared to conventional NOG mice, the CD4 T cells and B cells ratio was high and the CD8 killer T cell ratio was not increased, suggesting that acute GVHD was rather suppressed in this system. Moreover, the transplanted human T cells were shifted to a Th2 type based on the cytokine production profile (Fig 3C).

When the mice transplanted with healthy donor PBMC were immunized with the peptide vaccine CH401MAP or KLH, specific IgG was detected. In these mice, CD38 was expressed on almost all B cells, suggesting the development of plasma cells/plasmablasts [31,32]. We could not have identified functional DCs in this mouse system, but antigen-presentation by other myeloid cells or even by B cells might be a possibility if they express class II HLA. In adjuvant-injected mice, the plasma/plasmablast cell ratio was significantly increased in NOG-IL-4-Tg mice compared to mice that were not immunized, and it was also significantly higher than immunized NOG mice (S5 Fig). Therefore, it is possible that adjuvant injection in the hIL-4-abundant environment enhanced B cell proliferation, resulting in plasmablast differentiation. According to previous reports, CD38-positive plasmablasts are induced by IL-4-derived signals, although not all B cells expressing CD38 are mature plasma cells [30,33]. Under these conditions, secretion of antigen-specific IgG may be enhanced in PBMC-transplanted NOG-hIL-4-Tg mice. Moreover, naïve B cells cannot survive more than 4 weeks in vivo [34]. Because they do not have a long lifespan, if the cells are not stimulated to become memory B cells or plasma cells, then most of them cannot survive for 4 weeks in mice. This phenomenon does not conflict with our results because naïve B cells were not observed after 4 weeks. Although memory B cells have a longer lifespan, it is not as long as the plasma cell lifespan. Therefore, only plasma cells developed from memory or naïve B cells of PBMCs will remain in the spleen and BM. Moreover, the bone marrow of these mice cannot supply new naïve human B cells because of the absence of human hematopoietic stem cells. It may also enhance the superficial plasma/plasmablast cell ratio in these mice.

Plasmablasts usually accumulate in human BM, but they accumulated to a much greater extent in the spleen compared with the BM in PBMC-NOG-hIL-4-Tg mice (Fig 4C), which may have been due to the absence of human CXCL12-abundant reticular (CAR) cells [35]. Otherwise, because S1P/S1P_1_ signals transduced by erythrocytes elicit the migration of plasmablasts into the BM [36], reconstitution of human erythrocytes in NOG-hIL-4-Tg mice is feasible to resolve the issue. Collectively, the B cells in NOG-hIL-4-Tg mice developed into plasmablasts, but the final differentiation and migration achieved was less than would be expected in a normal human immune environment.

While HSC-transplanted immunodeficient mice induced a very poor antigen-specific IgG response, large improvement was observed in the system [37]. The improvements involved a human HLA-expressing mouse, which made it possible to induce antigen specific IgG production [14,15]. The mechanism enables the cognate interaction of human TCR and HLA expressed on the mouse APC. However, the HSCs of the donors must possess the same HLA type, which restricts the donor HLA type. Therefore, to achieve individualized medicine, a specific HLA-expressing mouse is not sufficient. From this perspective, the specific detection of IgG in NOG-IL-4-Tg is a prominent result of the development of a humanized mouse system.

Concerning the above argument, if the HLA type of the donor was determined and antibody production compared, then the prediction along with the algorithms shown in Table 1 may be improved. Although the reactivity of peptide has been predicted by several algorithms, the prediction is not always successful [38], potentially because of the difficulty associated with class II MHC prediction by the algorithms, the negative selection by auto-antigen recognition induced for the T cell repertoire, or the weakened immunity in the patients. Therefore, to predict the affinity of the peptide and MHC is not a completely adequate method to select a peptide for vaccination. Without determining the donor HLA, we can easily check the reactivity in the mouse similarly to the mixed lymphocyte reaction (MLR) before transfusion of the blood.

The difference between CH401MAP and KLH may have been caused by the high frequency of HER2-specific lymphocytes or the repetitive structure of the MAP peptide that cross-links the B cell receptor. Otherwise, the KLH molecule, which is rather large, may not be digested effectively by dendritic cells (DCs). The peptide reactivity was predicted by several algorithms, although high-score peptides are not always reactive with the native antigens [38]. Therefore, the efficacy of a vaccine in an individual patient cannot be predicted with these algorithms alone. However, using PBMC-NOG-hIL-4-Tg mice, the reactivity can be easily determined even if the donor HLA shows a low or undetermined reactivity score.

We observed large amounts of IgG in both NOG-IL-4-Tg and NOG mouse sera following PBS/Freund’s adjuvant immunization. While the adjuvant tended to enhance the number of plasma/plasmablast cells in NOG-IL-4-Tg but not in NOG mice (one NOG mouse that possessed an extremely high B cell ratio was removed from the analysis, the difference in plasma cell numbers was significant), the xenogeneic environment might induce antibodies against mouse tissues involving RBCs based on a previous report showing that huPBL engrafted into SCID mice could provoke strong T-dependent and polyclonal antibody responses to mouse erythrocyte antigens within 2–4 weeks post-engraftment of PBL [39]. We must clarify the efficiency of the antibody production against mice, and examine whether the specific antibody production could mimic the responses of the donors in NOG-IL-4-Tg. We will attempt to decrease the background of the ELISA by using new blockers such as those reported by Waritani et al. [40] for that.

Thus, when NOG mice were immunized with CH401MAP under the same conditions, the cross-reactivity against CH401MAP did not increase or the mice died from GVHD. These results supported the difficulty associated with the immunization and evaluation of antibody production in NOG mice, while many of the NOG-IL-4-Tg mice survived and showed enhanced CH401MAP-specific antibodies compared to the PBS/adjuvant control. Additionally, they possessed significant amounts of plasma/plasmablasts (S7 Fig).

As discussed above, the NOG-IL-4-Tg system is an attractive candidate as a vaccine design tool for patients. However, this system has limitations. First, the system is unable to induce the development of germinal centers in the spleen or lymph nodes. Consequently, this system cannot induce affinity maturation of each B cell clone. Another limitation, as mentioned above, is the potential induction of anti-mouse antibodies, which may mask the antigen-specific response. Finally, these mice cannot produce B cells continuously due to the absence of hematopoietic stem cell transplantation. Therefore, long-term observation following repetitive immunization is not possible. These limitations should be overcome to establish a sensitive system for the vaccination or treatment of individual patients.

Together, our results suggest that PBMC-NOG-IL-4-Tg mice can be vaccinated to secrete peptide-specific IgG antibodies. Moreover, class II restriction does not need to be accommodated in this system because most of the HDs had different HLA types and were still able to secrete peptide-specific IgG.

In conclusion, we succeeded in establishing a novel NOG-hIL-4-Tg mouse strain in which GVHD was suppressed following human PBMC transplantation and the production of antigen-specific IgG was maintained. This humanized mouse model may provide an effective tool for the design of human vaccines in the future.

## Supporting information

S1 Table The list of fluorochrome-labeled antibodies used for the FCM analysis. (DOCX)

S1 Fig Analysis of human lymphocytes in PBMC-NOG and PBMC-NOG-hIL-4-Tg mice. Three lines are shown: #3, #17 and #26. The lines were maintained, and the first progeny (N1) and second progeny (N2) were obtained. The plasma levels of human IL-4 were measured by ELISA. Mean values are shown above each bar, and the standard error is presented as error bars.
(PPTX)

S2 Fig Analysis of human lymphocytes in PBMC-NOG and PBMC-NOG-hIL-4-Tg mice. Spleen and BM cells from PBMC-NOG and PBMC-NOG-hIL-4-Tg mice were stained with fluorochrome-labeled antibodies and analyzed by FCM. Human PBMCs from HDs were used as controls. The typical patterns of each fraction are shown. Upper panels; T cell analysis. Lower panels; B cell analysis. For the T cell analysis, CD3+ cells were gated in the lymphoid cell fraction. These cells were further gated based on the CD45RA (naïve) and CD45RO (memory) populations. Each naïve or memory T cell fraction was further divided based on the expression of CD4 and CD8. For the B cell analysis, CD45+ cells in the lymphoid cell fraction were gated by CD19 (B cell) expression. These cells were divided into CD27+CD38- (memory) and CD38+ (plasmablast/plasma cell) B cell subsets. Transitional and naïve B cells were defined as the CD5+ and CD5- fractions, respectively, among CD27-CD38- cells.
(PPTX)

S3 Fig The profiles of human lymphocytes in PBMC-hIL-4-Tg-NOG mice following CH401MAP immunization. **A,** HD-PBMC and non-immunized/CH401MAP-immunized PBMC-NOG-hIL-4-Tg mouse-derived spleen cells and BM cells were stained with labeled antibodies and analyzed by FCM. Typical T cell profiles of the lymphocytes in HD PBMCs (left panels) and immunized PBMC-NOG-hIL-4-Tg spleen cells (spleen; middle panels) and BM cells (BM; right panels) are shown. The sets of surface markers analyzed are shown on the left side of the panels. Left panels; HD PBMCs. Middle panels with ‘Spleen’ label; PBMC-NOG-hIL-4-Tg spleen cells from non-immunized and immunized mice. Right panels with ‘BM’ label; PBMC-NOG-hIL-4-Tg BM. CD4+ T cells and CD4- T cells shown in the upper panels were further gated on CD4+ T cells (middle panels) and CD4- T cells (lower panels) and further analyzed by PD-1 (activated, exhausted) and CD25 (activated/Treg) expression. **B,** Typical B cell profiles in HD PBMC (left panels), non-immunized PBMC-NOG, non-immunized PBMC-NOG-hIL-4-Tg and immunized PBMC-NOG-hIL-4-Tg spleen cells (spleen; middle panels) and BM cells (BM; right panels) are shown. The sets of surface markers are shown on the left side of the panels. For the B cell analysis, CD45+ cells were gated on the lymphoid cell fraction. The gated cells were further gated based on CD19 (B cell) and CD5 (transitional/B1) expression (upper panels). The gated B cells were further divided by IgD (naïve B cell marker), CD21 (mature naïve, transitional 3 B cell marker), CD24 (immature, memory B cell marker), CD27 (memory B cell marker), CD38 (plasma/plasmablast marker) and CD138 (plasma cell marker) expression.
(PPTX)

S4 Fig The profiles of human lymphocytes in PBMC-hIL-4-Tg-NOG mice following CH401MAP and KLH immunization. Typical flow cytometric data shown in Fig 3A. Using the same method as described in S2 Fig, naïve/memory T cells and naïve/memory/transitional B cells and plasmablast/plasma cells were analyzed by FCM.
(PPTX)

S5 Fig Plasma/plasmablast cell ratio in the immunized NOG and NOG-IL-4-Tg mice. (A) The total spleen cell number and (B) the ratio of plasma cells (CD19+CD38+) in the spleen cells of the mice. (C) The number of plasma cells was calculated and is shown in the panels. No stimulation; mice without any treatment after PBMC transplantation. PBS; PBS/adjuvant-treated mice. CH401MAP; CH401MAP-immunized mice. All data were obtained from the mice used in Fig 3A, Fig 4F and S7 Fig. For the HD33-transplanted mice, spleen cells were collected immediately after the mouse died; the mouse number is 3. Mean values are indicated by bars. The Student’s *t*-test was performed, and the p-values are shown in the panels between the NOG and NOG-IL-4-Tg data. NOG: n = 3 for the PBS and CH401MAP-treatment. NOG-IL-4-Tg; n = 10 for the PBS and CH401MAP-treatment. For ‘No stimulation’, NOG; n = 6 and NOG-IL-4-Tg; n = 3. Total spleen cells were counted, and the total plasma cell number was calculated by the ratio and the number obtained for each analysis.
(PPTX)

S6 Fig Correlation of human IL-4 concentration and CH401MAP-specific IgG in the sera of NOG-IL-4-Tg mice. The correlation of the concentrations of hIL-4 (shown in Fig 1) and CH401-specific IgG (shown in Fig 4) in the sera of CH401 MAP-immunized NOG-IL-4-Tg mice was assessed (n = 10). The correlation coefficient and formula are shown in the scatter plot.
(PPTX)

S7 Fig Serum level of CH401MAP-specific IgG in NOG mice. NOG mice were immunized with CH401MAP or PBS using the same protocol as applied for NOG-IL-4-Tg. Five NOG mice were used: 2 of control (FCA only) and 3 CH401MAP-immunized mice. Two HDs were included, and PBMCs were used. One CH401MAP-immunized mouse died from GVHD before the analysis (day 13) and was labeled ND.
(PPTX)
