# Supplementary material for: NOG-hIL-4-Tg, a new humanized mouse model for producing tumor antigen-specific IgG antibody by peptide vaccination
Source: PLoS One. 2017 Jun 15;12(6):e0179239. doi: 10.1371/journal.pone.0179239 (PMC5472286; doi:10.1371/journal.pone.0179239)
Supplement: S1 Table — (DOCX) [file pone.0179239.s001.docx]

| Antibody | Clone | Company |
| --- | --- | --- |
| FITC anti-human CD3 | UCHT1 | BioLegend |
| PE/Cy7 anti-human CD3 | UCHT1 | BioLegend |
| APC anti-human CD4 | RPA-T4 | BioLegend |
| BrilliantViolet510^TM^ anti-humanCD4 | OKT4 | BioLegend |
| PE/Cy7 anti-human CD5 | UCHT2 | BioLegend |
| FITC anti-human CD8 | HIT8a | BioLegend |
| Pacific Blue^TM^ anti-human CD8a | RPA-T8 | BioLegend |
| APC anti-human CD16 | 3G8 | BioLegend |
| PE anti-human CD19 | HIB19 | BioLegend |
| APC/Cy7 anti-human CD19 | HIB19 | BioLegend |
| APC anti-human CD21 | FAB4909A | R＆D systems |
| PerCP/Cy5.5 anti-human CD24 | ML5 | BioLegend |
| PE anti-human CD25 | BC96 | BioLegend |
| PE/Cy7 anti-human CD25 | BC96 | BioLegend |
| PE anti-human CD27 | M-T271 | BD Bioscience |
| FITC anti-human CD33 | HIM3-4 | BioLegend |
| Alexa Fluor^®^700 anti-human CD38 | HIT2 | BioLegend |
| APC/Cy7 anti-human CD45 | HI30 | BioLegend |
| Pacific Blue^TM^ anti-human CD45 | HI30 | BioLegend |
| PE anti-human CD45RA | HI100 | BioLegend |
| APC anti-human CD45RA | HI100 | BioLegend |
| FITC anti-human CD45RO | UCHL1 | BioLegend |
| APC/Cy7 anti-human CD45RO | UCHL1 | BioLegend |
| PE anti-human CD56 | MEM-188 | BioLegend |
| PE/Cy7 anti-human CD56 | NCAM16.2 | BD Bioscience |
| FITC anti-human CD138 | DL-101 | eBioscience |
| PE anti-human CD197(CCR7) | G043H7 | BioLegend |
| PerCP/Cy5.5 anti-human CD279(PD-1) | EH12.2H7 | BioLegend |
| FITC anti-human IFNg | 4S.B3 | eBioscience |
| FITC anti-human IgD | IA6-2 | BD Bioscience |
| PE anti-human IL-4 | MP4-25D2 | eBioscience |

| Company | Address |
| --- | --- |
| BioLegend | 9727 Pacific Heights Blvd, San Diego, CA |
| R＆D systems Inc. | 614 McKinley Place NE, Minneapolis, MN |
| BD Bioscience | 1 Becton Drive, Franklin Lakes, NJ |
| eBioscience | 10255 Science Center Drive San Diego, CA |

**Supplemental Table 1** The list of fluorochrome-labeled antibodies used for the FCM analysis
