# Supplementary figures and images for: NOG-hIL-4-Tg, a new humanized mouse model for producing tumor antigen-specific IgG antibody by peptide vaccination
Source: PLoS One. 2017 Jun 15;12(6):e0179239. doi: 10.1371/journal.pone.0179239 (PMC5472286; doi:10.1371/journal.pone.0179239)

## Slide 1
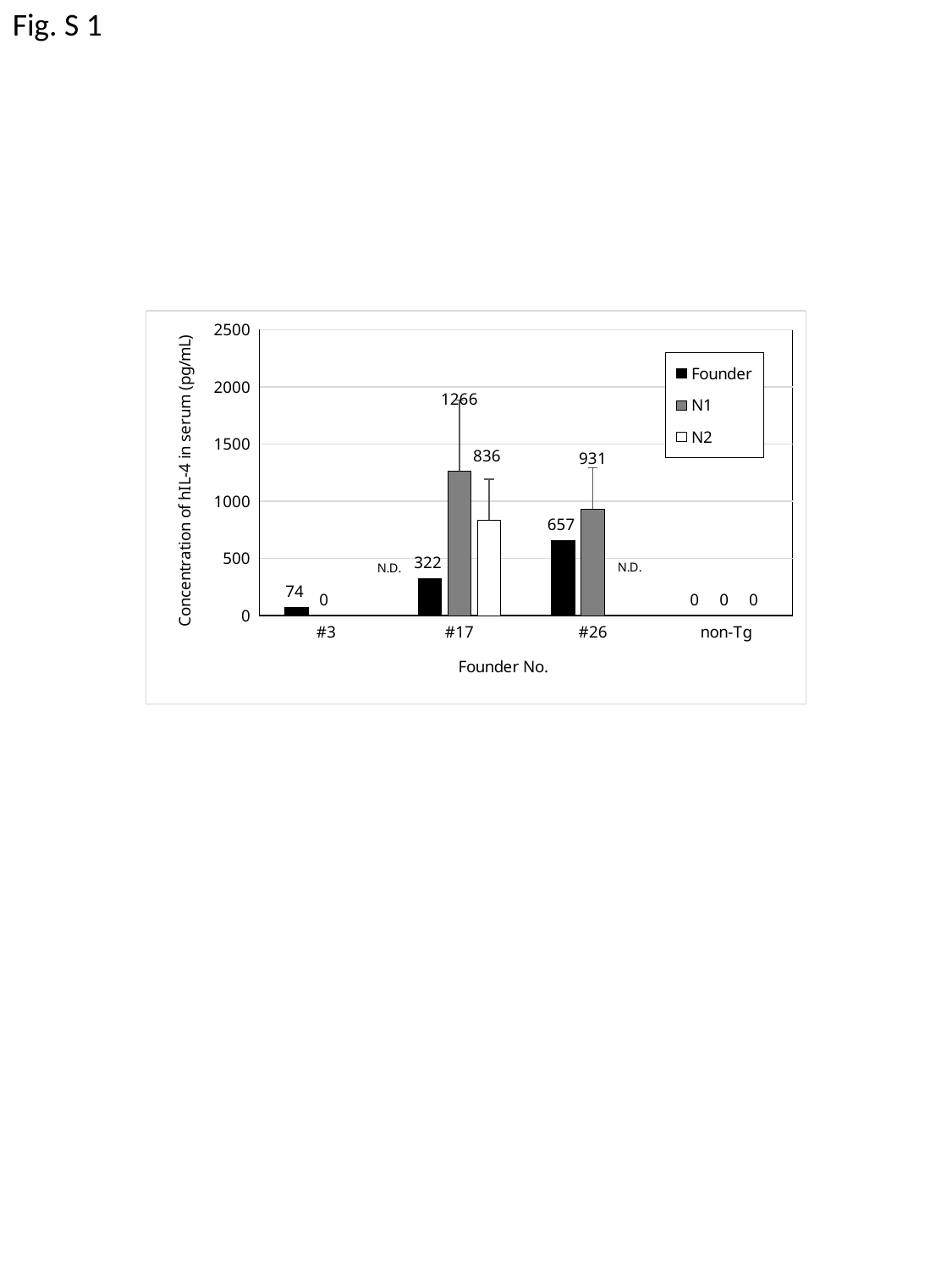

Fig. S 1
### Chart
| Category | Founder | N1 | N2 |
|---|---|---|---|
| #3 | 73.92 | 0.0 | None |
| #17 | 321.5 | 1265.75 | 836.0066666666665 |
| #26 | 656.5 | 931.2214285714285 | None |
| non-Tg | 0.0 | 0.0 | 0.0 |

Supplement: S1 Fig — Three lines are shown: #3, #17 and #26. The lines were maintained, and the first progeny (N1) and second progeny (N2) were obtained. The plasma levels of human IL-4 were measured by ELISA. Mean values are shown above each bar, and the standard error is presented as error bars. (PPTX) [file pone.0179239.s002.pptx]
