# Supplementary material for: NOG-hIL-4-Tg, a new humanized mouse model for producing tumor antigen-specific IgG antibody by peptide vaccination
Source: PLoS One. 2017 Jun 15;12(6):e0179239. doi: 10.1371/journal.pone.0179239 (PMC5472286; doi:10.1371/journal.pone.0179239)
Supplement: S2 Fig — Spleen and BM cells from PBMC-NOG and PBMC-NOG-hIL-4-Tg mice were stained with fluorochrome-labeled antibodies and analyzed by FCM. Human PBMCs from HDs were used as controls. The typical patterns of each fraction are shown. Upper panels; T cell analysis. Lower panels; B cell analysis. For the T cell analysis, CD3+ cells were gated in the lymphoid cell fraction. These cells were further gated based on the CD45RA (naïve) and CD45RO (memory) populations. Each naïve or memory T cell fraction was further divided based on the expression of CD4 and CD8. For the B cell analysis, CD45+ cells in the lymphoid cell fraction were gated by CD19 (B cell) expression. These cells were divided into CD27+CD38- (memory) and CD38+ (plasmablast/plasma cell) B cell subsets. Transitional and naïve B cells were defined as the CD5+ and CD5- fractions, respectively, among CD27-CD38- cells. (PPTX) [file pone.0179239.s003.pptx]

## Slide 1
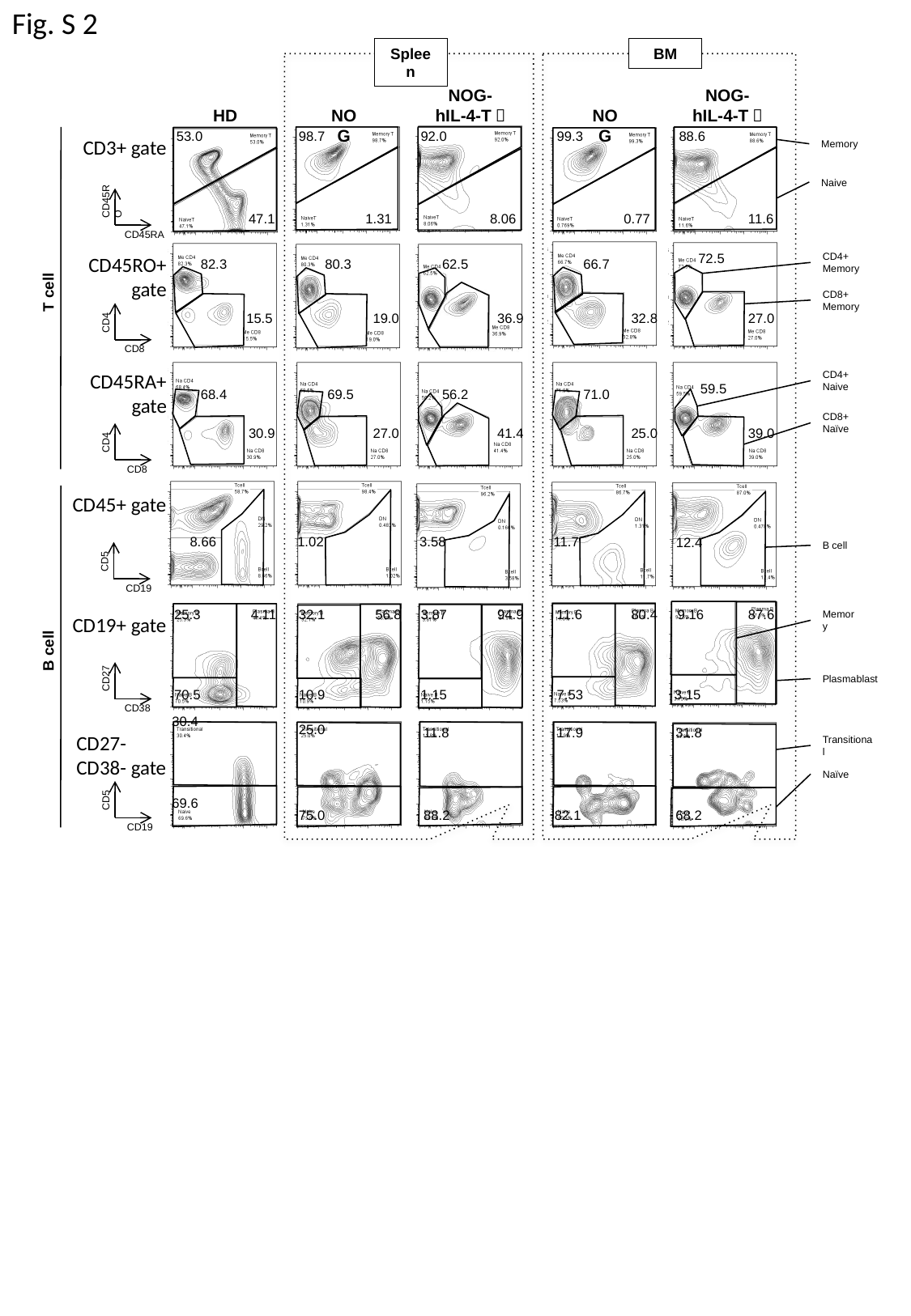

Fig. S 2
Spleen
BM
NOG-
hIL-4-Tｇ
NOG-
hIL-4-Tｇ
HD
NOG
NOG
53.0
98.7
92.0
99.3
88.6
CD3+ gate
Memory
Naive
CD45RO
47.1
1.31
8.06
0.77
11.6
CD45RA
72.5
CD4+
Memory
CD45RO+
gate
82.3
80.3
62.5
66.7
T cell
CD8+
Memory
15.5
19.0
36.9
32.8
27.0
CD4
CD8
CD45RA+
gate
CD4+
Naive
59.5
68.4
69.5
56.2
71.0
CD8+
Naïve
30.9
27.0
41.4
25.0
39.0
CD4
CD8
CD45+ gate
1.02
8.66
3.58
11.7
12.4
B cell
CD5
CD19
25.3
4.11
32.1
56.8
3.87
94.9
11.6
80.4
9.16
87.6
Memory
CD19+ gate
B cell
CD27
Plasmablast
10.9
1.15
7.53
3.15
70.5
CD38
30.4
25.0
11.8
17.9
31.8
CD27-
CD38- gate
Transitional
Naïve
CD5
69.6
75.0
88.2
82.1
68.2
CD19
