# Supplementary material for: NOG-hIL-4-Tg, a new humanized mouse model for producing tumor antigen-specific IgG antibody by peptide vaccination
Source: PLoS One. 2017 Jun 15;12(6):e0179239. doi: 10.1371/journal.pone.0179239 (PMC5472286; doi:10.1371/journal.pone.0179239)
Supplement: S3 Fig — A, HD-PBMC and non-immunized/CH401MAP-immunized PBMC-NOG-hIL-4-Tg mouse-derived spleen cells and BM cells were stained with labeled antibodies and analyzed by FCM. Typical T cell profiles of the lymphocytes in HD PBMCs (left panels) and immunized PBMC-NOG-hIL-4-Tg spleen cells (spleen; middle panels) and BM cells (BM; right panels) are shown. The sets of surface markers analyzed are shown on the left side of the panels. Left panels; HD PBMCs. Middle panels with ‘Spleen’ label; PBMC-NOG-hIL-4-Tg spleen cells from non-immunized and immunized mice. Right panels with ‘BM’ label; PBMC-NOG-hIL-4-Tg BM. CD4+ T cells and CD4- T cells shown in the upper panels were further gated on CD4+ T cells (middle panels) and CD4- T cells (lower panels) and further analyzed by PD-1 (activated, exhausted) and CD25 (activated/Treg) expression. B, Typical B cell profiles in HD PBMC (left panels), non-immunized PBMC-NOG, non-immunized PBMC-NOG-hIL-4-Tg and immunized PBMC-NOG-hIL-4-Tg spleen cells (spleen; middle panels) and BM cells (BM; right panels) are shown. The sets of surface markers are shown on the left side of the panels. For the B cell analysis, CD45+ cells were gated on the lymphoid cell fraction. The gated cells were further gated based on CD19 (B cell) and CD5 (transitional/B1) expression (upper panels). The gated B cells were further divided by IgD (naïve B cell marker), CD21 (mature naïve, transitional 3 B cell marker), CD24 (immature, memory B cell marker), CD27 (memory B cell marker), CD38 (plasma/plasmablast marker) and CD138 (plasma cell marker) expression. (PPTX) [file pone.0179239.s004.pptx]

## Slide 1
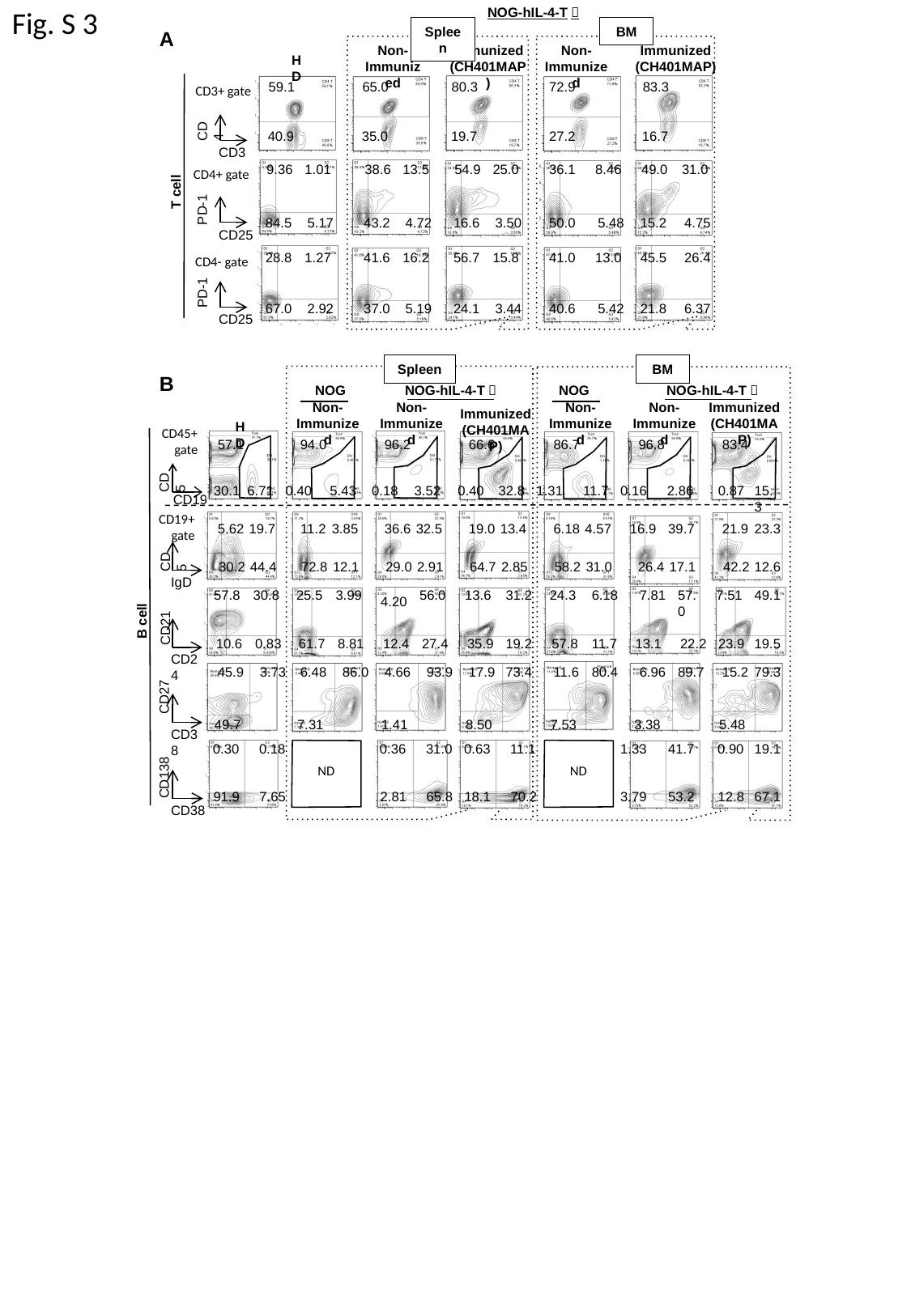

Fig. S 3
NOG-hIL-4-Tｇ
Spleen
BM
A
Non-
Immunized
Immunized
(CH401MAP)
Non-
Immunized
Immunized
(CH401MAP)
HD
59.1
65.0
80.3
72.9
83.3
CD3+ gate
CD4
40.9
35.0
19.7
27.2
16.7
CD3
9.36
1.01
38.6
13.5
54.9
25.0
36.1
8.46
49.0
31.0
CD4+ gate
T cell
PD-1
84.5
5.17
43.2
4.72
16.6
3.50
50.0
5.48
15.2
4.75
CD25
28.8
1.27
41.6
16.2
56.7
15.8
41.0
13.0
45.5
26.4
CD4- gate
PD-1
67.0
2.92
37.0
5.19
24.1
3.44
40.6
5.42
21.8
6.37
CD25
Spleen
BM
B
NOG
NOG-hIL-4-Tｇ
NOG
NOG-hIL-4-Tｇ
Non-
Immunized
Non-
Immunized
Non-
Immunized
Non-
Immunized
Immunized
(CH401MAP)
Immunized
(CH401MAP)
HD
CD45+
gate
57.1
94.0
96.2
66.6
86.7
96.8
83.4
CD5
30.1
6.71
0.40
5.43
0.18
3.52
0.40
32.8
1.31
11.7
0.16
2.86
0.87
15.3
CD19
CD19+
gate
5.62
19.7
11.2
3.85
36.6
32.5
19.0
13.4
6.18
4.57
16.9
39.7
21.9
23.3
CD5
IgD
30.2
44.4
72.8
12.1
29.0
2.91
64.7
2.85
58.2
31.0
26.4
17.1
42.2
12.6
57.8
30.8
25.5
3.99
56.0
13.6
31.2
24.3
6.18
7.81
57.0
7.51
49.1
4.20
CD21
CD24
B cell
10.6
0.83
61.7
8.81
12.4
27.4
35.9
19.2
57.8
11.7
13.1
22.2
23.9
19.5
45.9
3.73
6.48
86.0
4.66
93.9
17.9
73.4
11.6
80.4
6.96
89.7
15.2
79.3
CD27
CD38
49.7
7.31
1.41
8.50
7.53
3.38
5.48
0.30
0.18
0.36
31.0
0.63
11.1
1.33
41.7
0.90
19.1
ND
ND
CD138
91.9
7.65
2.81
65.8
18.1
70.2
3.79
53.2
12.8
67.1
CD38
