# Supplementary material for: NOG-hIL-4-Tg, a new humanized mouse model for producing tumor antigen-specific IgG antibody by peptide vaccination
Source: PLoS One. 2017 Jun 15;12(6):e0179239. doi: 10.1371/journal.pone.0179239 (PMC5472286; doi:10.1371/journal.pone.0179239)
Supplement: S4 Fig — Typical flow cytometric data shown in Fig 3A. Using the same method as described in S2 Fig, naïve/memory T cells and naïve/memory/transitional B cells and plasmablast/plasma cells were analyzed by FCM. (PPTX) [file pone.0179239.s005.pptx]

## Slide 1
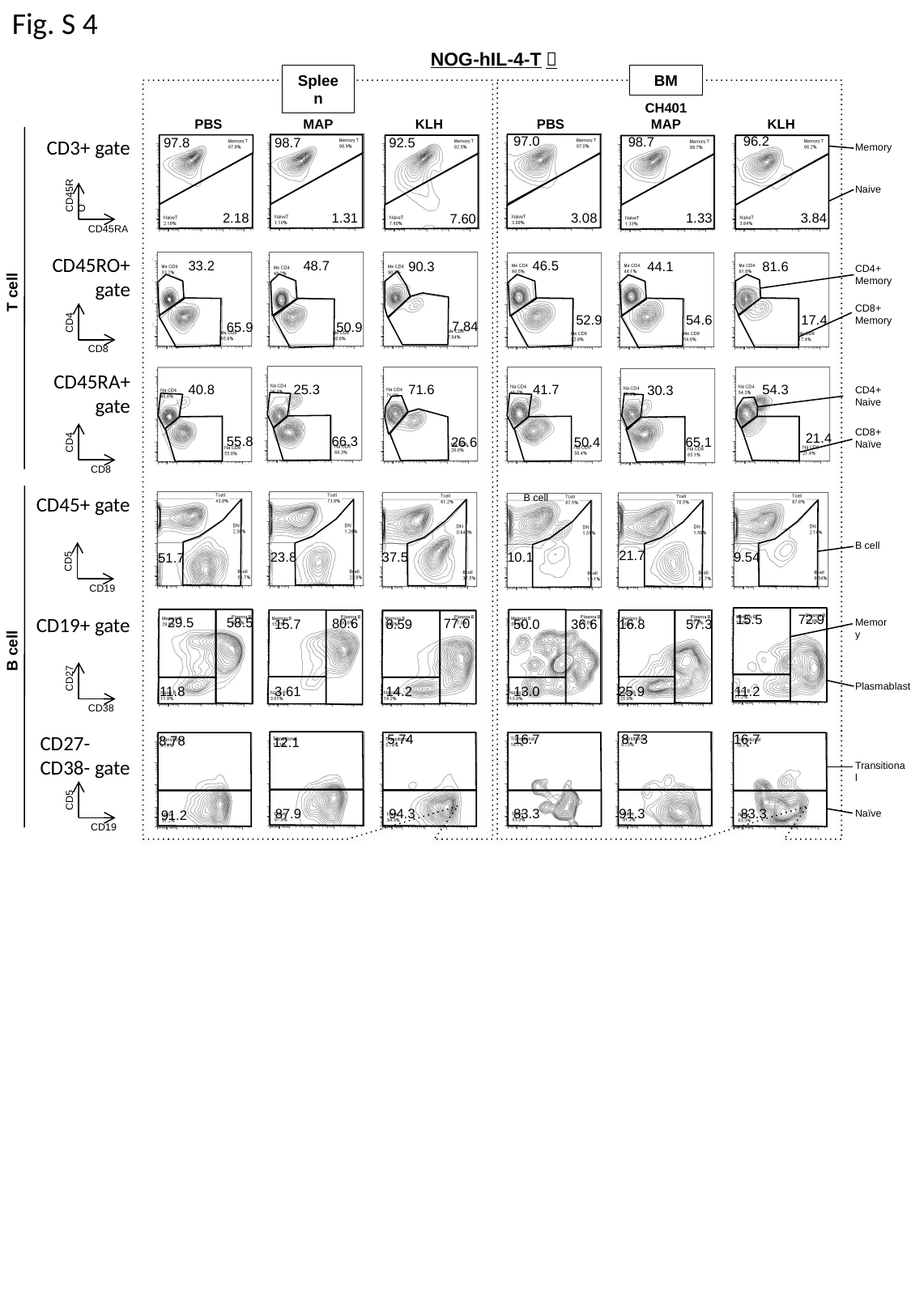

Fig. S 4
NOG-hIL-4-Tｇ
Spleen
BM
CH401
MAP
CH401
MAP
PBS
KLH
PBS
KLH
96.2
97.0
98.7
97.8
98.7
92.5
CD3+ gate
Memory
Naive
CD45RO
2.18
1.31
3.08
1.33
3.84
7.60
CD45RA
CD45RO+
gate
33.2
48.7
46.5
90.3
44.1
81.6
CD4+
Memory
T cell
CD8+
Memory
CD4
52.9
54.6
17.4
7.84
65.9
50.9
CD8
CD45RA+
gate
40.8
25.3
71.6
41.7
54.3
30.3
CD4+
Naive
CD8+
Naïve
21.4
CD4
55.8
66.3
50.4
26.6
65.1
CD8
CD45+ gate
B cell
B cell
21.7
23.8
37.5
10.1
9.54
51.7
CD5
CD19
72.9
15.5
CD19+ gate
29.5
58.5
77.0
80.6
50.0
36.6
Memory
15.7
8.59
16.8
57.3
B cell
CD27
Plasmablast
13.0
11.8
3.61
14.2
25.9
11.2
CD38
CD27-
CD38- gate
5.74
16.7
8.73
16.7
8.78
12.1
Transitional
CD5
94.3
83.3
91.3
83.3
87.9
Naïve
91.2
CD19
