# Supplementary material for: NOG-hIL-4-Tg, a new humanized mouse model for producing tumor antigen-specific IgG antibody by peptide vaccination
Source: PLoS One. 2017 Jun 15;12(6):e0179239. doi: 10.1371/journal.pone.0179239 (PMC5472286; doi:10.1371/journal.pone.0179239)
Supplement: S5 Fig — (A) The total spleen cell number and (B) the ratio of plasma cells (CD19+CD38+) in the spleen cells of the mice. (C) The number of plasma cells was calculated and is shown in the panels. No stimulation; mice without any treatment after PBMC transplantation. PBS; PBS/adjuvant-treated mice. CH401MAP; CH401MAP-immunized mice. All data were obtained from the mice used in Fig 3A, Fig 4F and S7 Fig. For the HD33-transplanted mice, spleen cells were collected immediately after the mouse died; the mouse number is 3. Mean values are indicated by bars. The Student’s t-test was performed, and the p-values are shown in the panels between the NOG and NOG-IL-4-Tg data. NOG: n = 3 for the PBS and CH401MAP-treatment. NOG-IL-4-Tg; n = 10 for the PBS and CH401MAP-treatment. For ‘No stimulation’, NOG; n = 6 and NOG-IL-4-Tg; n = 3. Total spleen cells were counted, and the total plasma cell number was calculated by the ratio and the number obtained for each analysis. (PPTX) [file pone.0179239.s006.pptx]

## Slide 1
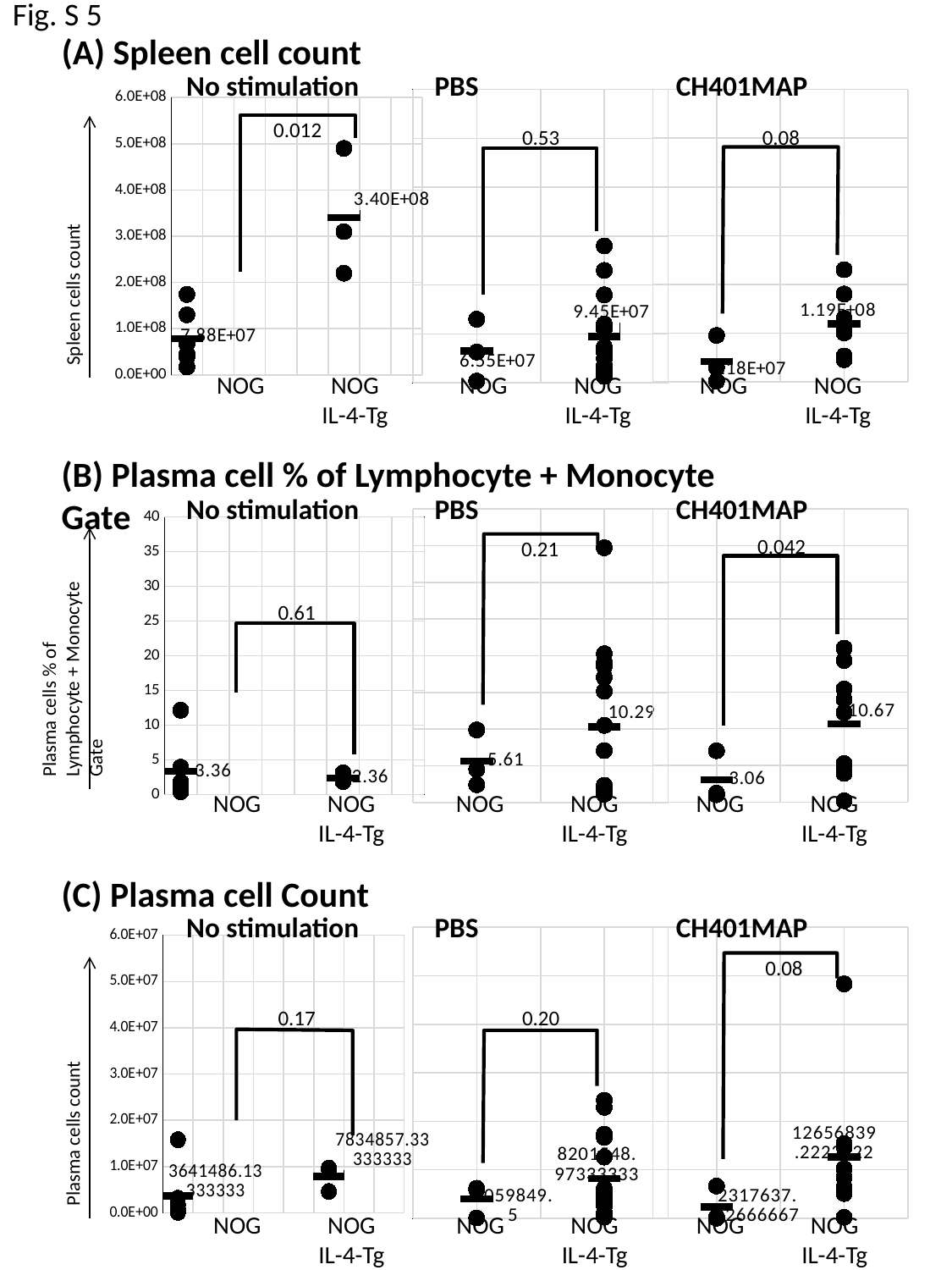

Fig. S 5
(A) Spleen cell count
No stimulation
PBS
CH401MAP
### Chart
| Category | |
|---|---|
### Chart
| Category | |
|---|---|
### Chart
| Category | |
|---|---|0.012
0.08
0.53
Spleen cells count
NOG
NOG
IL-4-Tg
NOG
NOG
IL-4-Tg
NOG
NOG
IL-4-Tg
(B) Plasma cell % of Lymphocyte + Monocyte Gate
No stimulation
PBS
CH401MAP
### Chart
| Category | |
|---|---|
### Chart
| Category | |
|---|---|
### Chart
| Category | |
|---|---|0.042
0.21
0.61
Plasma cells % of
Lymphocyte + Monocyte Gate
NOG
NOG
IL-4-Tg
NOG
NOG
IL-4-Tg
NOG
NOG
IL-4-Tg
(C) Plasma cell Count
No stimulation
PBS
CH401MAP
### Chart
| Category | |
|---|---|
### Chart
| Category | |
|---|---|
### Chart
| Category | |
|---|---|0.08
0.20
0.17
Plasma cells count
NOG
NOG
IL-4-Tg
NOG
NOG
IL-4-Tg
NOG
NOG
IL-4-Tg
