# Supplementary material for: NOG-hIL-4-Tg, a new humanized mouse model for producing tumor antigen-specific IgG antibody by peptide vaccination
Source: PLoS One. 2017 Jun 15;12(6):e0179239. doi: 10.1371/journal.pone.0179239 (PMC5472286; doi:10.1371/journal.pone.0179239)
Supplement: S6 Fig — The correlation of the concentrations of hIL-4 (shown in Fig 1) and CH401-specific IgG (shown in Fig 4) in the sera of CH401 MAP-immunized NOG-IL-4-Tg mice was assessed (n = 10). The correlation coefficient and formula are shown in the scatter plot. (PPTX) [file pone.0179239.s007.pptx]

## Slide 1
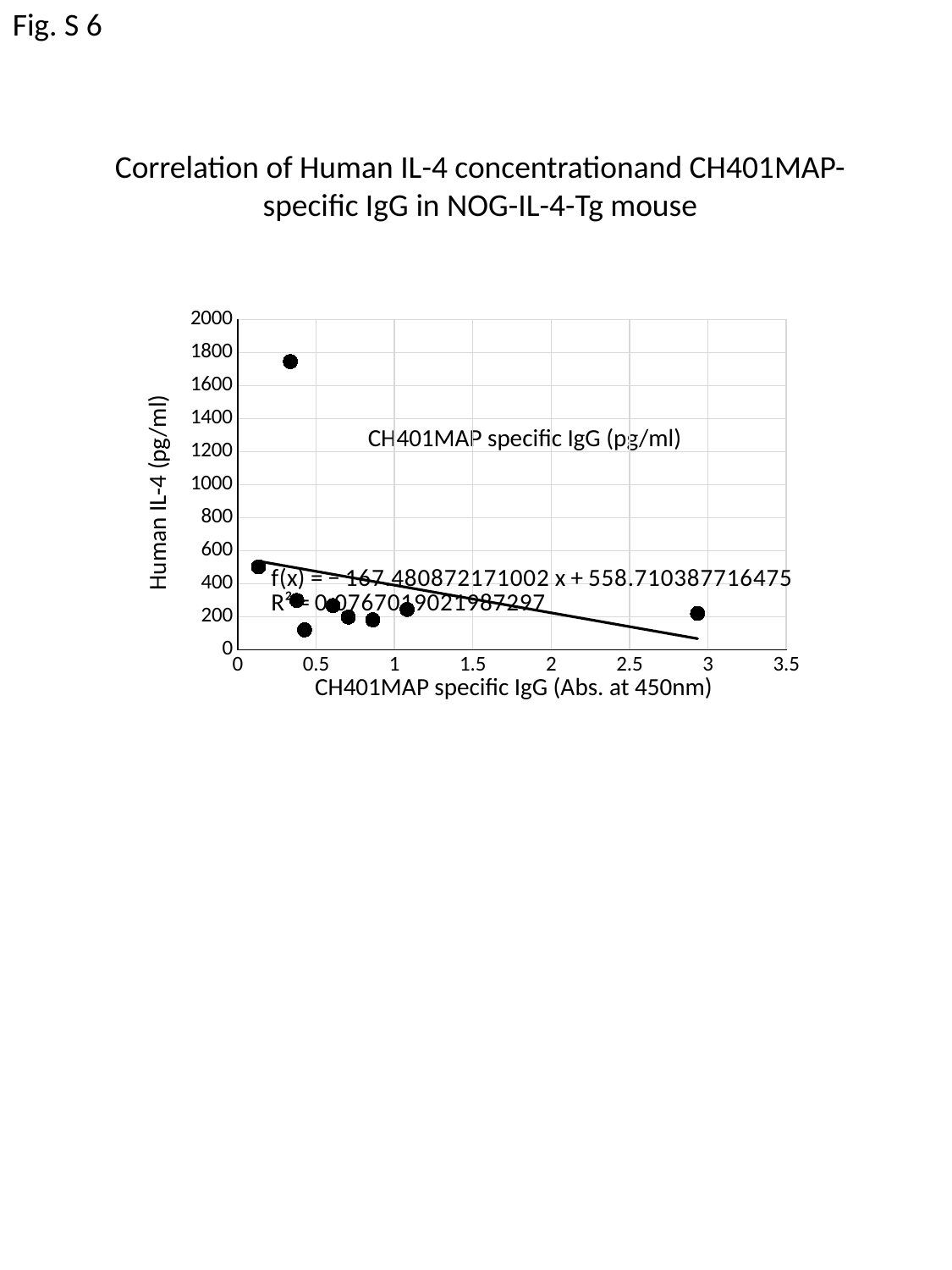

Fig. S 6
Correlation of Human IL-4 concentrationand CH401MAP-specific IgG in NOG-IL-4-Tg mouse
### Chart
| Category | |
|---|---|CH401MAP specific IgG (pg/ml)
Human IL-4 (pg/ml)
CH401MAP specific IgG (Abs. at 450nm)
