# Supplementary material for: NOG-hIL-4-Tg, a new humanized mouse model for producing tumor antigen-specific IgG antibody by peptide vaccination
Source: PLoS One. 2017 Jun 15;12(6):e0179239. doi: 10.1371/journal.pone.0179239 (PMC5472286; doi:10.1371/journal.pone.0179239)
Supplement: S7 Fig — NOG mice were immunized with CH401MAP or PBS using the same protocol as applied for NOG-IL-4-Tg. Five NOG mice were used: 2 of control (FCA only) and 3 CH401MAP-immunized mice. Two HDs were included, and PBMCs were used. One CH401MAP-immunized mouse died from GVHD before the analysis (day 13) and was labeled ND. (PPTX) [file pone.0179239.s008.pptx]

## Slide 1
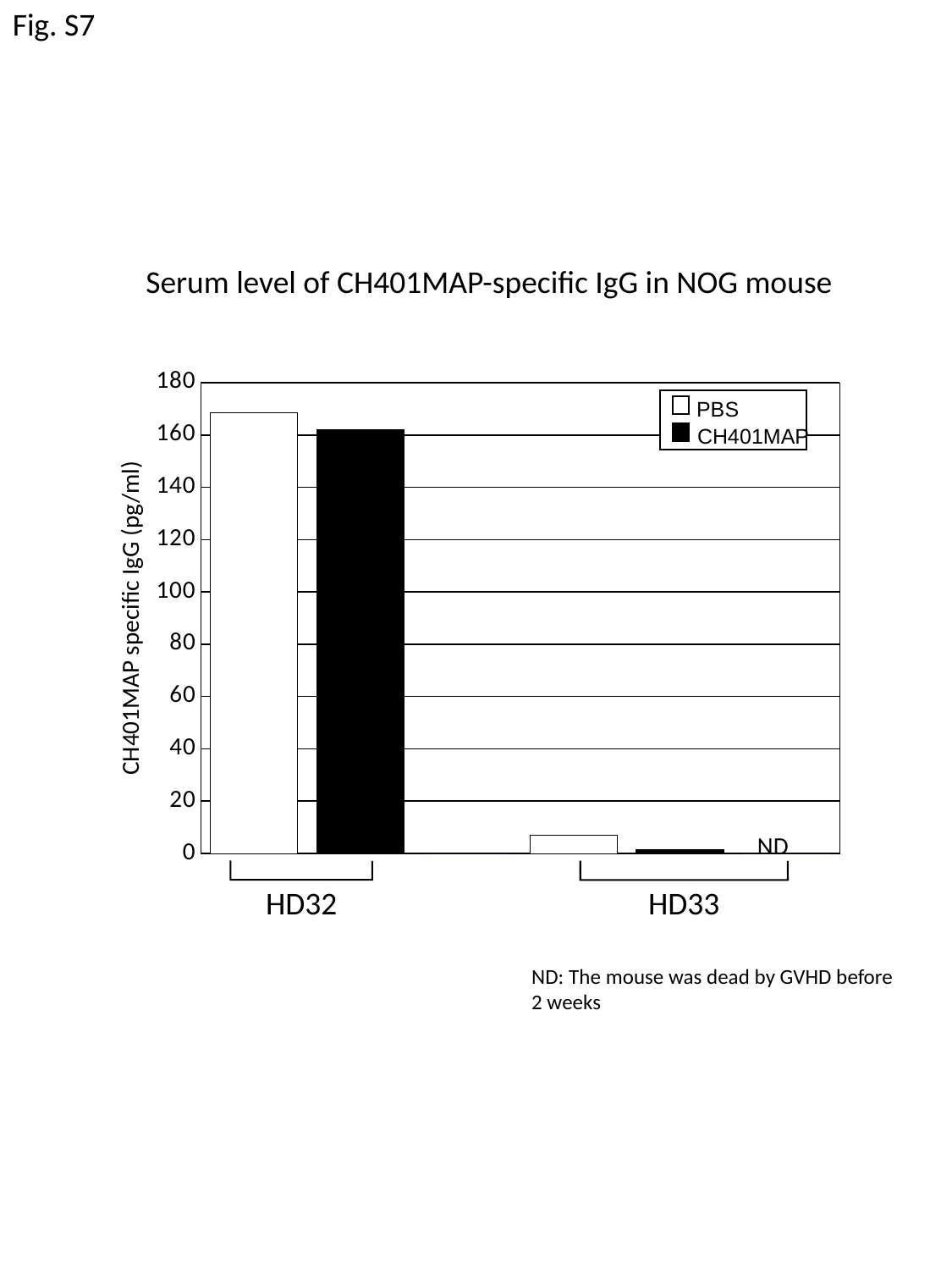

Fig. S7
Serum level of CH401MAP-specific IgG in NOG mouse
### Chart
| Category | |
|---|---|
| 1.0 | 168.6 |
| 2.0 | 162.0 |
| 3.0 | 0.0 |
| 4.0 | 6.827999999999993 |
| 5.0 | 1.532 |
| 6.0 | 0.0 |PBS
CH401MAP
CH401MAP specific IgG (pg/ml)
ND
HD32
HD33
ND: The mouse was dead by GVHD before 2 weeks
